# Anion-Exchange Blue Perovskite Quantum Dots for Efficient Light-Emitting Devices

**DOI:** 10.3390/nano12223957

**Published:** 2022-11-10

**Authors:** Wei-Kuan Hung, Yi-Hsun Tseng, Chun-Cheng Lin, Sih-An Chen, Chih-Hung Hsu, Chen-Feng Li, Yen-Ju Chen, Zong-Liang Tseng

**Affiliations:** 1 Department of Electro-Optical Engineering, National Taipei University of Technology, Taipei 10608, Taiwan; 2 Department of Mathematic and Physical Sciences, General Education, R.O.C. Air Force Academy, Kaohsiung 820009, Taiwan; 3 Giant-Tek Corporation, Miaoli 35048, Taiwan; 4 Department of Electronic Engineering, Ming Chi University of Technology, New Taipei 243303, Taiwan

**Keywords:** perovskite, quantum-dot (QD), nanoparticle (NP), light-emitting diodes (LED), energy efficiency

## Abstract

In this study, blue perovskite quantum dots (PQDs) were prepared using didodecyldimethylammonium bromide (DDAB), which can passivate surface defects caused by the loss of surface ligands and reduce particle size distribution. After the passivation of DDAB, blue CsPbCl_x_Br_3−x_ PQDs dispersed in n-octane produced a more compact and uniform PQD thin film than the non-passivated ones. The resulting device showed a stabile lifetime, and an EL peak of 470 nm and a maximum EQE of 1.63% were obtained at an operating voltage of 2.6 V and a current density of 0.34 mA/cm^2^. This work aims to provide a simple method to prepare blue-emitting PQDs and high-performance PQD-based light-emitting devices.

## 1. Introduction

Recently, blue LEDs have been gradually developed and are widely used in various fields such as agricultural growth [1,2,3,4,5], white LED development [6,7] and biomedical research [8,9,10,11]. The increasingly prevalent medical aesthetic industry has also invested in the study of blue LEDs for teeth whitening and dental care treatment via photodynamic therapy [12], which enables a high-intensity blue light to inhibit the growth of dental plaque and germs on the material surface [13]. Furthermore, with the compensation with the use of infrared light, it can be utilized in pain control [14].

In 2014, the first light-emitting diode (LED) was developed using perovskite quantum dots as the light-emitting layer [15,16,17], indicating the current research trend. The attractiveness of perovskite is due to its incredibly narrow FWHM [18,19,20], extremely high color purity in the band, tunable wavelengths for a wide range of applications, low cost for easy fabrication and excellent performance in photoluminescence quantum yields (PLQYs) [21,22], causing PQD-based LEDs (PQLEDs) to rapidly develop in the past few years. The external quantum efficiency (EQE) of the PQLEDs have had several major breakthroughs for green, red and near-infrared regions, but the blue PQLEDs exhibit a relatively slow development [23].

Song et al. first proposed the blue PQLEDs using CsPbCl_x_Br_3−x_ PQDs synthesized by a traditional hot-injection method, achieving an EQE of 0.07% [24]. Subsequently, various strategies have been presented to improve the device performance of the blue PQLEDs [25]. The surface–ligand exchange methods were commonly used to decrease the surface defeat of PQDs, improve stability and enhance charge injection [26,27,28,29,30,31]. In addition, the anion exchange methods provide the Cl^−^ anions to pristine green CsPbBr_3_ PQDs to tune the emission wavelength and bandgap of PQDs. The didodecyldimethylammonium chloride (DDAC) ligands were employed to supply DDA^+^ and Cl^−^ anions for CsPbBr_3_ PQDs, achieving a maximum EQE of 1.9% and a maximum luminance of 35 cd/m^2^ at a blue-shifted 490 nm emission peak [32]. Conjugated amidine ligands such as acetamidine hydrochloride (AMCl), pentanimidamide hydrochloride (PMCl) and benzamidine hydrochloride (BMCl) are introduced into pristine green CsPbBr_3_ PQDs to synthesize CsPbCl_x_Br_3−x_ PQDs, achieving a maximum EQE of 2.3%, and a maximum luminance of 205 cd/m^2^ at a blue-shifted 479 nm emission peak [33]. Similarly, the direct synthesis of CsPbCl_x_Br_3−x_ PQDs using mixture ligands of DDAC and didodecyldimethylammonium bromide (DDAB) showed high-quality PQDs with fine-tuning of the blue-shifted emission wavelength, resulting in an EQE of 0.44% at 470 nm and 0.86% at 480 nm [34]. In contrast to the blue-shifted PQDs, the deep-blue CsPbCl_x_Br_3−x_ PQDs exchanged by the Br^−^ anions to obtain the red-shifted blue-emission wavelength have not yet been reported.

Here, the synthesis of the CsPbCl_x_Br_3−x_ PQDs solution was post-treated by using didecyldimethylammonium bromide (DDAB) for surface–ligand exchange and Br/Cl anion ratios to obtain improved PLQYs, crystallization and a tunable blue-emission wavelength from 452 to 474 nm. In addition, the uniform distribution of the particle size can also be obtained after the DDAB modification, leading to smooth and high-quality PQD films coated on substrates. The DDAB method for CsPbCl_x_Br_3−x_ PQDs were optimized by controlling the concentration of DDAB in the ligand exchange solution. Additionally, we demonstrated highly bright and efficient DDAB-modified CsPbCl_x_Br_3−x_-based PQLEDs, achieving an EQE approaching 1.63% at 470 nm. Therefore, we believe that our works may provide a simple method for low-cost blue PQDs and PQLED to expand the promising potential of PQDs.

## 2. Materials and Methods

### 2.1. Synthesis of CsPbBr_3_ QD Solution

A total of 2 mL of octadecene (ODE; 90%; Sigma Aldrich, St. Louis, MO, USA), 0.9 mL of oleic acid (OA; 90%; Sigma Aldrich) and 0.2442 g of caesium carbonate (Cs_2_CO_3_; 99.99%, Acros Organics, Geel, Belgium) powder were added to a 50 mL sample vial and were stirred at 120 °C for 1 h to introduce the residual oxygen from the solution. Afterwards, the solution was heated to 150 °C and stirred for 30 min, appearing clear and colorless. Finally, the solution was cooled down to 100 °C and was reserved for the follow-up hot-injection method. In total, 0.01, 0.02, 0.03 and 0.04 mmol of didecyldimethylammonium bromide (DDAB; 98%; Sigma Aldrich) were added to 0.6 mL of oleic acid (OA) and 2 mL of toluene (Toluene; 99.7%; ACS grade; Honeywell, Charlotte, NC, USA) and shattered with an ultrasonic cleaner until appearing clear for the follow-up ligand exchange reaction for DDAB*1, DDAB*2, DDAB*3 and DDAB*4, respectively. In total, 10 mL of octadecene (ODE), 0.1104 g of lead bromide (PbBr_2_; 99.998%; Alfa Aesar, Tewksbury, MA, USA) powder and 0.99 g n-octyl ammonium chloride powder were added to a 50 mL sample vial and stirred at 120 °C for 1 h to remove the dissolved oxygen from the solution. Then, 0.6 mL of oleic acid (OA) and 0.6 mL of n-octylamine (OCTAm; 99%; Sigma Aldrich) were added to the solution under nitrogen filling and were heated to 170 °C. After that, 0.6 mL of oleic acid (OA) and 0.6 mL of n-octylamine were added under nitrogen filling and were heated to 170 °C. Afterwards, 0.8 mL of a cesium oleate precursor was added to the solution and kept at 170 °C for 1 min of reaction. Finally, the ligand exchange solution was added at room temperature under nitrogen flow and stirred for 5 min before the ice bath step to obtain the crude solution of CsPbCl_x_Br_3−x_ PQDs. The synthesized crude solution of PQDs was added into the centrifuge tube, and the bottom sediment was collected by centrifugation at 12,000 rpm for 10 min; then, hexane (Hexane; 95%; Sigma-Aldrich) and ethyl acetate (ETOAC; AR (ACS); MACHEREY-NAGEL, Dueren, Germany) were injected into the solution and centrifuged twice. Finally, QD powder was dispersed in octane (Octane; 98+%; Alfa Aesar).

### 2.2. Device Fabrication

The patterned ITO substrate was wet-cleaned and treated with O_2_ plasma. PEDOT: PSS (Al 4083) solution was filtered with a hydrophobic PVDF syringe filter (0.44 μm) and spinning coated at 5000 rpm for 20 s prior to annealing at 150 °C for 10 min. Afterwards, a VB-FNPD solution was spin-coated at 2000 rpm for 20 s, and then heated to 100 °C for 5 min prior to heating to 170 °C for 30 min (double annealing). After that, the ITO substrate coated with PEDOT:PSS and VB-FNPD was placed in a nitrogen glove box, where the QD solution was extracted and filtered with a hydrophilic PVDF syringe filter (0.22 μm); then, it was spin coated at 1500 rpm for 20 s and was baked at 60 °C for 1 min. Subsequently, CNT2T, LiF and Al were deposited on the substrate in the vapor deposition chamber at 5 × 10^−6^ torr. Their sets of film thickness and deposition rate were 60 nm at 0.3 Å/s, 0.7 nm at 0.3 Å/s and 100 nm at 3 Å/s, respectively. Finally, the devices were encapsulated with a glass cover and the UV-curing adhesive under the UV lamp for 5 min.

### 2.3. Measuring Instruments

The PL spectra were measured by a fluorescence spectrophotometer (F-7000, Hitachi, Tokyo, Japan). The TRPL decays were obtained using a HORIBA FluoroMax Plus spectrofluorometer. EL characteristics were recorded using an LQ-100R spectrometer (Enlitech, Kaohsiung, Taiwan) with a computer-controlled integrating sphere and a Keysight B2901A source meter. The XRD patterns were analyzed using an AFM system (INNOVA AFM, Bruker, Billerica, MS, USA) and an X-ray diffractometer (D8 advance, Bruker), respectively. The TEM images were performed with the JEOL (Peabody, MA, USA) JEM-2100 microscope. TEM samples were prepared by 5–10 µL of purified QD solution was then drop-cast on the TEM copper grid and allowed to dry completely. Elemental analysis data were acquired via Energy-dispersive X-ray spectroscopy (EDS) combinated with a JEOL JEM-2100 microscope. The size distribution of the QDs was measured using a dynamic light scattering spectrophotometer (ELSZ-2000ZS, OTSUKA, Tokyo, Japan).

## 3. Results and Discussion

**Figure 1:**
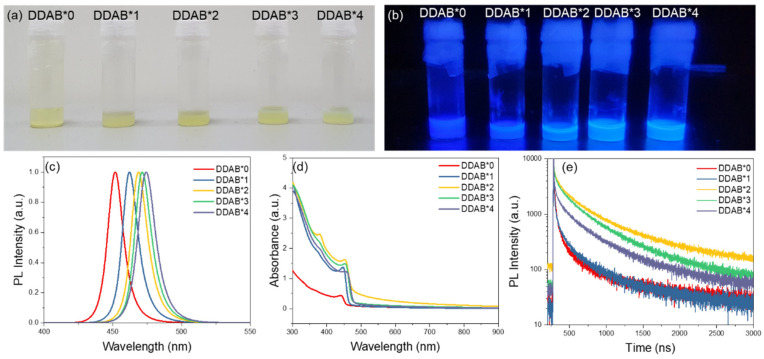
Photo images under (**a**) ambient light and (**b**) UV light of 365 nm; (**c**) PL, (**d**) absorption and (**e**) TRPL spectra of CsPbCl_x_Br_3−x_ PQDs with different DDAB concentrations.

As shown in Figure 1a,b, five solutions of PQDs with different ratios were obtained by adding 0 mmol to 4 mmol of DDAB to the crude solution of CsPbCl_x_Br_3−x_ (denoted as DDAB*0, DDAB*1, DDAB*2, DDAB*3 and DDAB*4). The photoluminescence (PL) of the PQD solutions after DDAB modification showed a noticeable redshift, as shown in Figure 1c. The peaks of emission were 452 nm, 462 nm, 469 nm, 471 nm and 474 nm, when the amounts of DDAB were added from 0 mmol to 4 mmol. During the anion exchange reaction, the Cl^−^ ions in CsPbCl_x_Br_3−x_ PQDs were replaced, and the overall proportion of Br^−^ ions increased, thereby changing the emission wavelength. The reason for the redshift in the spectrum could be attributed to the increase in Br^−^ ions with the increase in DDAB concentration. Likewise, the absorption edges of the DDAB-modified PQDs showed a similar redshift trend, as shown in Figure 1d. Figure 1e shows the time-resolved photoluminescence (TRPL) of PQDs. With the data, the exciton average lifetime (τavg) can be fitted from the TRPL decay curves using the single exponential decay function (summarized in Table S1), exhibiting a near-unity radiative channel [35]. When the crude solution of CsPbCl_x_Br_3−x_ was modified by DDAB, the ligand may have been exchanged between DDA^+^ and n-octylamine. The DDA^+^ cations may have passivated the surface defects of the PQDs, which improved the radiative recombination and shortened the exciton lifetime [30,36]. The DDAB*2 showed the best exciton lifetime, which was three times higher than that of the sample without DDAB.

**Figure 2:**
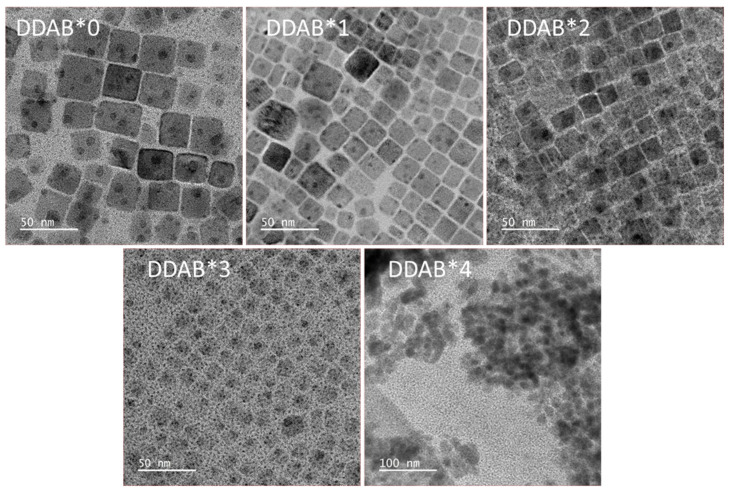
TEM images of CsPbCl_x_Br_3−x_ PQDs treated by different DDAB concentrations.

Figure 2 shows CsPbCl_x_Br_3−x_ PQDs with different DDAB concentrations. The particles showed a cubic shape in the DDAB*0, DDAB*1, DDAB*2 and DDAB*3 samples. The large-range distribution of particle size can be seen in DDAB*0. After the DDAB modification, the FWHMs of the diameter distribution were gradually decreased with the increase in DDAB concentration, as shown in Figure S1. The FWHM of the size distribution of the PQDs became more and narrower with the increasing DDAB, which means the uniformity of the PQD sizes was improved by the DDAB modification. The particle sizes also decreased when the DDAB concentration increased (Figure S1). DDAB*0, DDAB*1 and DDAB*2 clearly showed a cubic particle shape. On the contrary, when the DDAB dosage was gradually increased, the cubic shape of the DDAB*3 and DDAB*4 samples became unclear. It may be attributed to the fact that the Pb/Cs element ratio with an increasing DDAB concentration deviated from the ideal chemical stoichiometric ratio of one, as shown in Table S2. In addition, it is worth mentioning that serious aggregations could be seen in the DDAB*4 samples due to the high Pb/Cs element ratio of 2.29, causing us to be unable to analyze the particle size of the DDAB*4 sample (Figure S1).

**Figure 3:**
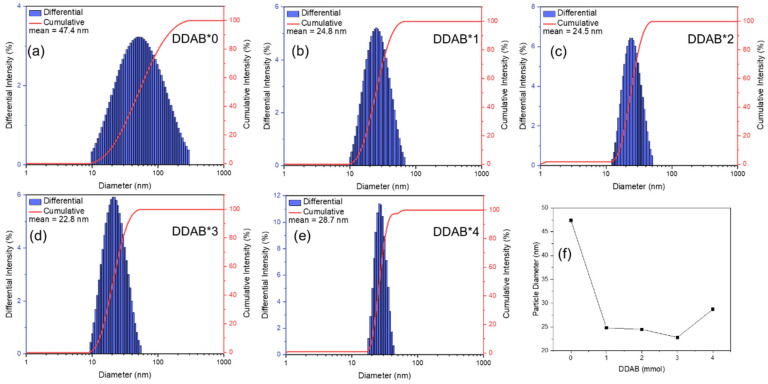
The diameter distribution of CsPbCl_x_Br_3−x_ PQDs treated by (**a**) DDAB*0, (**b**) DDAB*1, (**c**) DDAB*2, (**d**) DDAB*3, and (**e**) DDAB*4. (**f**) The mean diameters of PQDs with different DDAB concentrations.

On the other hand, the TEM images were obtained using the dry samples on the copper mesh (TEM sample preparation is in the Supporting Information), implying that the aggregation may be interfered by other factors such as the surface tension of the solvent (octane) after evaporation. To evaluate the aggregation degree in the PQD dispersions, dynamic light scattering (DLS) was used to confirm the particle size in the PQD dispersions, again. Figure 3a–e shows the particle size analysis of the CsPbCl_x_Br_3−x_ PQDs measured by dynamic light scattering. The average particle diameters decreased with the increase in DDAB concentrations. The reason may be inferred that the higher ratio would result in a higher exchange rate for DDA^+^ ligands on the surfaces of CsPbCl_x_Br_3−x_ PQDs, and stabilize the growth of PQDs to achieve homogeneity [37]. When DDAB increased, the concentration of DDA^+^ increased, which restricted the growth of the PQDs with steric hindrance and resulted in smaller sized PQDs [33,38]. In addition, one can also observe the decreased FWHM of the diameter distribution after DDAB passivation, indicating a more uniform particle size. The trenches of the diameter distributions obtained using TEM (Figure S1) and DLS were similar, but the decreased FWHM of the diameter distribution in DDAB*4 could be observed, indicating the DDA^+^ presence in the PQD dispersions. Furthermore, the average particle diameter of DDAB*4 was slightly larger than those of DDAB*1, DDAB*2 and DDAB*3, as shown in Figure 3f. It is attributed to the Br^−^ overdose, leading to an abnormal particle size and the aggregation (TEM image of DDAB*4).

**Figure 4:**
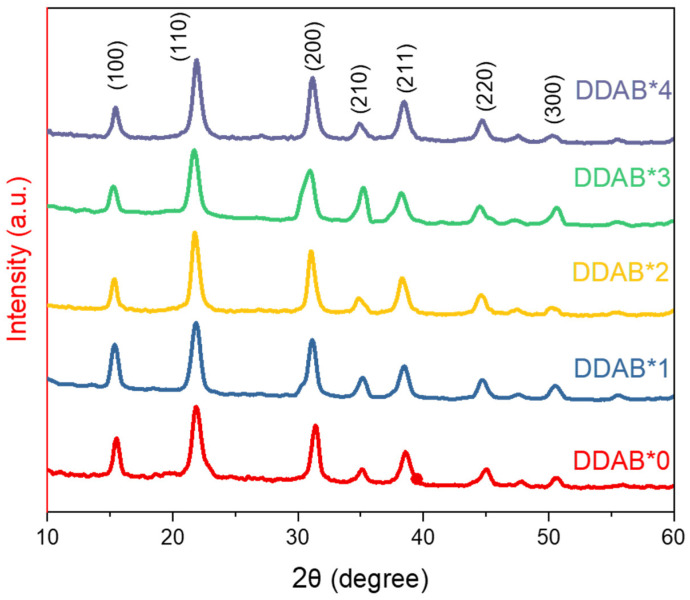
XRD pattern of CsPbCl_x_Br_3−x_ PQDs treated by different DDAB concentrations.

To further verify the crystalline phase of the PQDs, X-ray diffractometer confirmed that all the PQD samples showed obvious cubic diffraction peaks, as shown in Figure 4. It indicated that the crystalline phase was stable, and no impurity phase was generated due to the addition of DDAB. The EDS analysis (Table S2) showed that the Cl^−^/Br^−^ ratio gradually decreased with the increase in the DDAB concentration (higher Br^−^ anion rate). According to Bragg’s Law (nλ = 2D Sinθ, where λ is the wavelength of the incident X-ray, n is the diffraction order, D is d spacing described as the distance between planes of atoms at a given diffraction peaks, and θ is the included angle between the incident X-ray and lattice plane), the radius of the Br^−^ ion is larger than that of the Cl^−^ ion, so d-spacing increases with a decreasing Cl^−^/Br^−^ ratio when λ is constant. The d-spacing decreases with increasing in θ. Therefore, the (100) and (110) peaks that shifted to a larger angle (Table 1) could be ascribed to the increased Cl^−^/Br^−^ ratio, implying the redshift PL emission (Figure 1c). In addition, the Pb^2+^/Cs^+^ ratio gradually increased from 1.16 (DDAB*0) to 2.33 (DDAB*4) with the increase in the DDAB concentration, deviating the stoichiometric ratio of the perfect CsPbX3 (Pb^2+^/Cs^+^ = 1). It agreed with the TEM image of DDAB*4, implying that the non-cubic shape was caused by the wrong chemical component.

**Figure 5:**
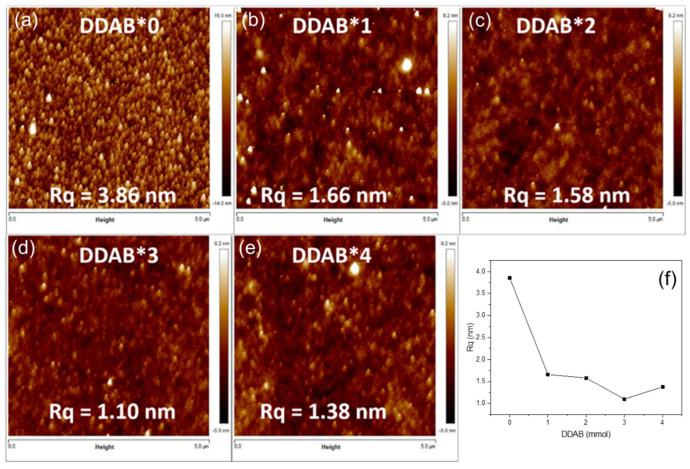
AFM images of the films coated with CsPbCl_x_Br_3−x_ PQDs treated by (**a**) DDAB*0, (**b**) DDAB*1, (**c**) DDAB*2, (**d**) DDAB*3, and (**e**) DDAB*4. (**f**) The root mean square roughness (Rq) of PQD films coated with different DDAB concentrations.

In the solution process, the PQD uniformity of the coated film had a great impact on the quality of the PQD films. The roughness of the PQD films can cause an extra leakage current and can degrade the overall electrical characteristics. From the AFM measurements shown in Figure 5, the films fabricated by PQDs without adding DDAB measured Rq = 3.86 nm. With the addition of DDAB, the measured Rq of the quantum dot films ranged from 1.66 nm to 1.10 nm. DDAB enabled the PQDs to produce a more compact stack of film and a more uniform film, due to the uniform particle distribution (Figure 2 and Figure 3). Furthermore, the larger film roughness in DDAB*4 than those of DDAB*1, DDAB*2 and DDAB*3 is attributed to the particle aggregation, which agreed with the TEM image of DDAB*4 and Figure 3e.

**Figure 6:**
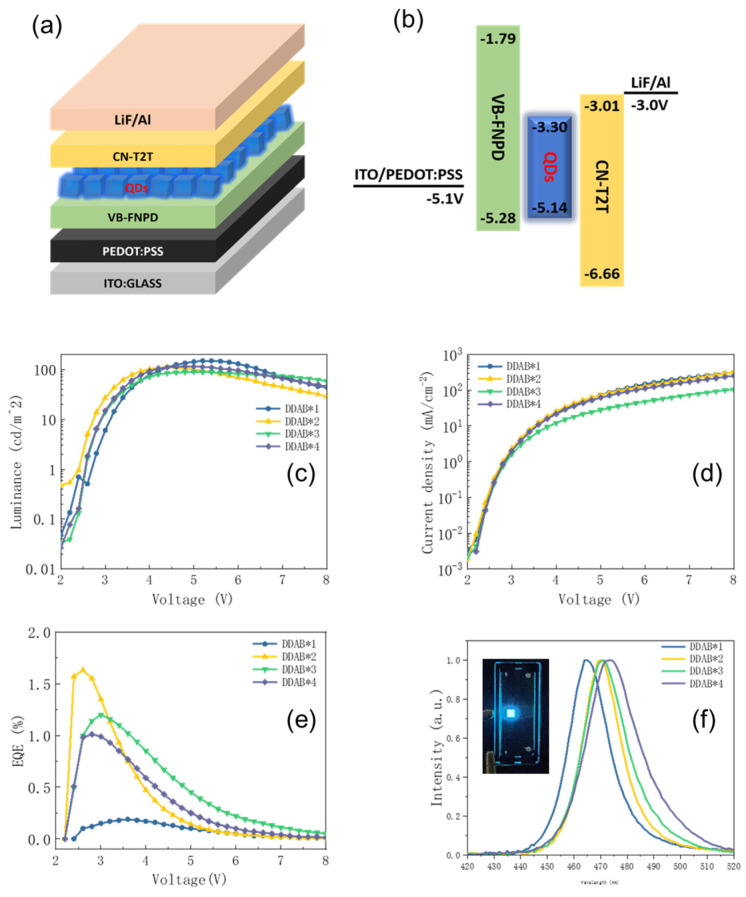
(**a**) Schematic diagram of structure and (**b**) energy level diagram of the blue PQLED device; (**c**) luminance−voltage curve, (**d**) current−voltage curve, (**e**) EQE−voltage curve and (**f**) EL spectra of the PQLED device based on CsPbCl_x_Br_3−x_ PQDs treated by different DDAB concentrations. The inset in (**f**) is the photo of DDAB*2 Device.

Figure 6a,b show the structure and the energy level of the PQLED device based on the DDAB-modified PQDs. The DDAB*0 device could be prepared due to the high roughness of the emission layer (Figure 5). Figure 6c shows the L-V curve, which shows that the DDAB*2 device had the best turn-on voltage (operating voltage at 1 cd/m^2^) of around 2.5 V, implying the appropriate DDAB concentration. Figure 6d shows the I-V curve, in which a similar curve can be found among all four devices due to the same device structure. Therefore, the device structure is suitable to be fabricated for the DDAB-modified PQDs. In the EQE-V curves shown in Figure 6e, the DDAB*2 device had a more remarkable performance, and exhibited a maximum EQE of 1.63% at 2.6 V and 0.34 mA/cm^2^, which is attributed to the longer exciton lifetime (Figure 1e). The EL spectra in Figure 6f were consistent with the previous PL results with the same redshift trend, and the EL emission was from 465 nm to 475 nm.

## 4. Conclusions

In the study, we demonstrated a strategy to prepare blue CsPbCl_x_Br_3−x_ PQDs and the corresponding PQLEDs. The surface passivation of CsPbCl_x_Br_3−x_ PQDs with DDAB reduced surface defects, leading to a longer exciton lifetime. DDAB also provided Br^−^ ions for deep blue CsPbCl_x_Br_3−x_ PQDs with a PL emission at 452 nm to redshift to 474 nm. In addition, the DDAB-modified CsPbCl_x_Br_3−x_ PQDs showed a better roughness to form high uniform and compact films. As a result, the high-performance blue PQLED with a maximum EQE of 1.63% was obtained. This study may pave a new path for the stable production of blue PQLEDs.

## Figures and Tables

**Table 1:** XRD peak positions of CsPbCl_x_Br_3−x_ PQDs treated by different DDAB concentrations.

	(hkl)	(100)	(110)	(200)	(210)	(211)	(220)	(300)
DDAB*0	2θ(degree)	15.50	21.92	31.38	35.08	38.68	44.96	50.73
	d-spaceing(Å)	5.712085	4.052382	2.84877	2.556268	2.326202	2.013899	1.798275
DDAB*1	2θ(degree)	15.37	21.86	31.13	35.28	38.43	44.63	50.48
	d-spaceing(Å)	5.760104	4.063371	2.871079	2.542231	2.34076	2.028879	1.806594
DDAB*2	2θ(degree)	15.32	21.81	31.03	34.78	38.28	44.58	50.28
	d-spaceing(Å)	5.778791	4.072576	2.880105	2.57763	2.349587	2.031038	1.813311
DDAB*3	2θ(degree)	15.30	21.78	31.03	34.78	38.28	44.53	50.13
	d-spaceing(Å)	5.7863	4.078119	2.880105	2.57763	2.349587	2.033203	1.818385
DDAB*4	2θ(degree)	15.26	21.72	30.93	35.28	38.23	44.53	50.63
	d-spaceing(Å)	5.801377	4.089251	2.88919	2.542231	2.352546	2.033203	1.801592

## Supplementary Materials

The following supporting information can be downloaded at: https://www.mdpi.com/article/10.3390/nano12223957/s1. Figure S1: The distribution of particle diameters evaluated from TEM images of DDAB*0, DDAB*1, DDAB*2 and DDAB*3; Figure S2: The operational lifetime of LED devices; Figure S3: CsPbClxBr3−x PQDs preparation scheme; Table S1: The fitting exciton lifetime of PQDs treated by different DDAB concentration; Table S2: Element ratio analysis of PQDs treated by different DDAB concentration.
