# Supplementary material for: Anion-Exchange Blue Perovskite Quantum Dots for Efficient Light-Emitting Devices
Source: Nanomaterials (Basel). 2022 Nov 10;12(22):3957. doi: 10.3390/nano12223957 (PMC9693500; doi:10.3390/nano12223957)
Supplement: Supplementary file 1 [file nanomaterials-12-03957-s001.zip › nanomaterials-2000251-supplementary.pdf]

Supplementary Materials

Anion-Exchange Blue Perovskite Quantum Dots for Efficient Light-Emitting Devices

Wei-Kuan Hung [1,*], Yi-Hsun Tseng [1], Chun-Cheng Lin [2], Sih-An Chen [1,2], Chih-Hung Hsu [3], Chen-Feng Li [1],
Yen-Ju Chen [4] and Zong-Liang Tseng [4,*]

[1] Department of Electro-Optical Engineering, National Taipei University of Technology, Taipei 10608, Taiwan
[2] Department of Mathematic and Physical Sciences, General Education, R.O.C. Air Force Academy, Kaohsiung 820009, Taiwan
[3] Giant-Tek Corporation, Miaoli 35048, Taiwan
[4] Department of Electronic Engineering, Ming Chi University of Technology, New Taipei 243303, Taiwan
* Correspondence: wkhung@ntut.edu.tw (W.-K.H.); zltseng@mail.mcut.edu.tw (Z.-L.T.)

Table S1. The fitting exciton lifetime of PQDs treated by different DDAB concentration.

	DDAB*0	DDAB*1	DDAB*2	DDAB*3	DDAB*4
**τ** (ns)	6.63	8.50	23.69	18.76	9.19

Table S2. Element ratio analysis of PQDs treated by different DDAB concentration.

	Pb	Cs	Cl	Br	Pb/Cs	Cl/Br
DDAB*0	24.9%	21.5%	37.5%	16.1%	1.16	2.33
DDAB*1	24.2%	20.5%	32.4%	22.9%	1.18	1.41
DDAB*2	23.4%	15.8%	35.2%	25.6%	1.48	1.38
DDAB*3	26.0%	17.4%	20.4%	36.2%	1.50	0.56
DDAB*4	22.0%	9.6%	11.3%	57.2%	2.29	0.20

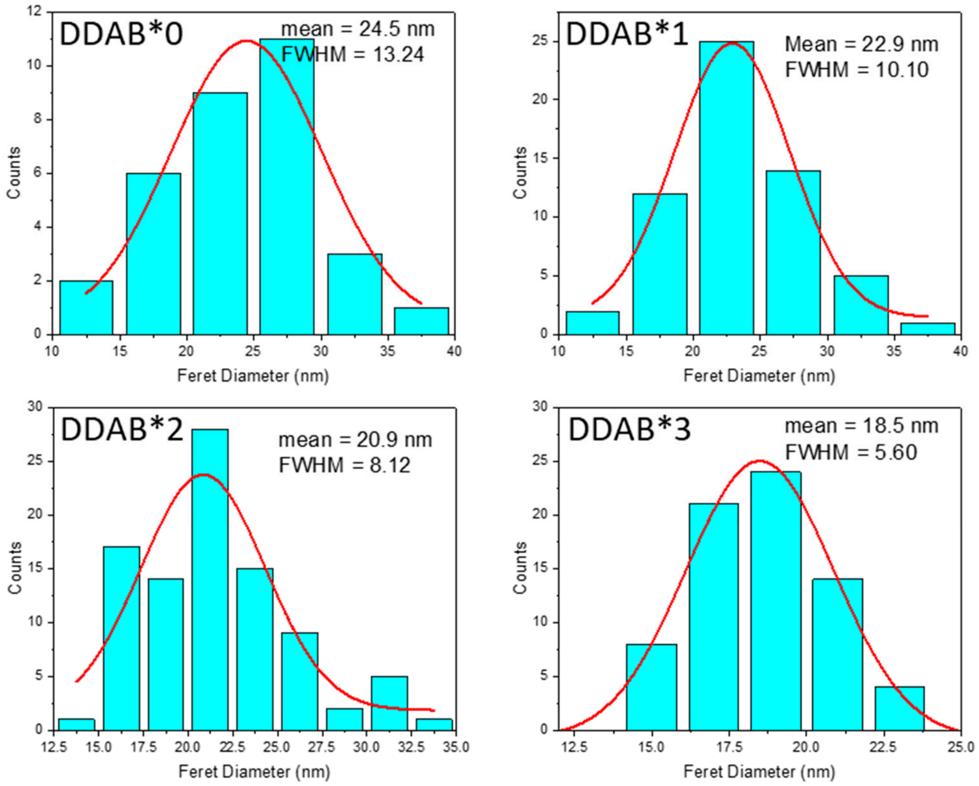

Figure S1. The distribution of particle diameters evaluated from TEM images of DDAB*0, DDAB*1, DDAB*2, and DDAB*3. ImageJ software was utilized for Figure 2 by "Threshold Color" with appropriate contrast and brightness to evaluate the diameter of the $CsPbCl_xBr_{3-x}$ PQDs in using "Analyze Particle" functions.

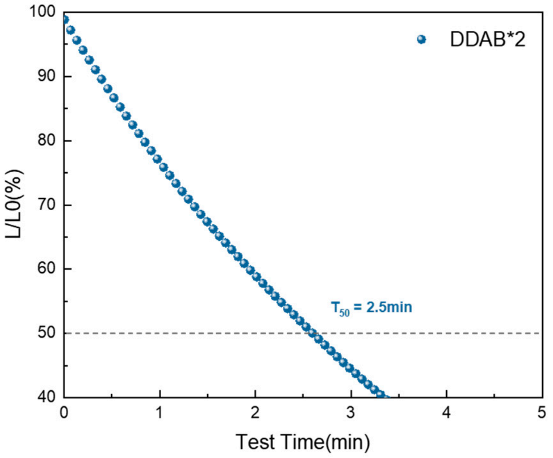

Figure S2. The operational lifetime of LED devices

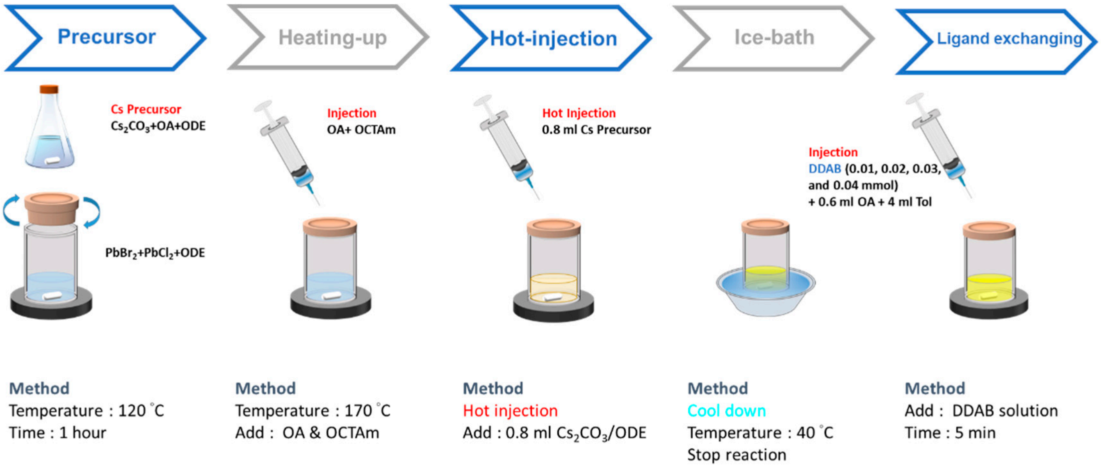

Figure S3. $CsPbCl_xBr_{3-x}$ PQDs preparation scheme
